# The road to young‐onset dementia diagnosis: Findings from the Joint Solutions Project

**DOI:** 10.1002/alz.70538

**Published:** 2025-08-12

**Authors:** Priscilla Tjokrowijoto, Clare Beard, Debbie Stange, Nathan M D'Cunha, Jade Cartwright, Naomi Moylan, Adrienne Withall, Brian Draper, Theresa Scott, Muireann Irish, Robyn Lewis, Kelly Atkins, Claire Goodlet, Elissa Burton, Rachael Cvejic, Karen Glennen, Daniel Schweitzer, Monica Cations, Samantha M Loi

**Affiliations:** ^1^ Neuropsychiatry Centre Royal Melbourne Hospital Parkville VIC Australia; ^2^ College of Education Psychology and Social Work Flinders University Adelaide SA Australia; ^3^ Wesley College Melbourne VIC Australia; ^4^ Centre for Ageing Research and Translation University of Canberra Bruce Australia; ^5^ School of Health Sciences University of Tasmania Launceston Tasmania Australia; ^6^ Brightwater Care Group Inglewood WA Australia; ^7^ School of Psychology Faculty of Science University of New South Wales Sydney NSW Australia; ^8^ Discipline of Psychiatry and Mental Health University of New South Wales Sydney NSW Australia; ^9^ School of Psychology Faculty of Health Medicine and Behavioural Sciences University of Queensland Queensland Australia; ^10^ Brain and Mind Centre University of Sydney Camperdown NSW Australia; ^11^ School of Psychological Sciences Monash University Clayton VIC Australia; ^12^ Curtin School of Allied Health Faculty of Sciences Curtin University Perth WA Australia; ^13^ enAble Institute Curtin University Perth WA Australia; ^14^ National Centre of Excellence in Intellectual Disability Health Faculty of Medicine and Health University of New South Wales Sydney NSW Australia; ^15^ Mater Hospital Brisbane QLD Australia; ^16^ Department of Psychiatry University of Melbourne Parkville VIC Australia

**Keywords:** Australia, barriers, care pathway, diagnosis, lived experience, opportunities, young‐onset dementia

## Abstract

**INTRODUCTION:**

Diagnosis of young‐onset dementia (YOD) is often delayed due to atypical presentations and lack of awareness. This study explored diagnostic experiences in Australia.

**METHODS:**

This Joint Solutions sub‐study employed a mixed‐methods approach. Surveys and focus groups targeted people with YOD, caregivers, and clinicians across Australia. Quantitative data were analyzed using descriptive statistics and comparisons, while qualitative data underwent thematic analysis.

**RESULTS:**

There were 313 participants, mostly female and nearly half representing lived experience. The average age at symptom onset was 55.8 years, and at diagnosis, 57.9 years. Positive aspects included timely diagnoses and involvement of specialized clinicians. Clinicians emphasized comprehensive history‐taking and a multifaceted diagnostic approach. Geographical barriers, variations in diagnosis delivery, and caregiver education needs were noted.

**DISCUSSION:**

This study highlights improved diagnostic timelines but ongoing barriers in YOD knowledge and equitable access to care. Raising awareness, improving clinician education, and streamlining referral processes are essential.

**Highlights:**

- Timeliness of younger‐onset dementia diagnosis appears to have improved in Australia.
- Access to care varies in availability and quality, with no standardized pathways.
- Knowledge of younger‐onset dementia is lacking in both healthcare and the community.
- Comprehensive history‐taking and a multifaceted diagnostic approach are crucial.
- Clinician sensitivity is valued, balanced with tailored education on diagnosis.

## 1 BACKGROUND

Dementia is a general term for the loss of cognitive abilities (e.g., memory, language, and executive function) that is severe enough to interfere with one's ability to perform daily activities, and can result from various conditions. Young‐onset dementia (YOD) is characterized by the onset of symptoms before the age of 65 years.^1^ While older‐onset dementia is predominantly associated with Alzheimer's disease pathology,^2^ YOD typically encompasses a broader range of underlying neurodegenerative conditions and secondary dementias, and its initial presentation may not necessarily involve memory problems.^1^ For example, behavioral and personality changes are often the initial concerns in frontotemporal dementia. Approximately 28,900 people are living with YOD in Australia, accounting for 7% of all dementia cases.^3^

The path to a YOD diagnosis is commonly associated with delays and misdiagnoses.^4^, ^5^, ^6^ Receiving a diagnosis of dementia is particularly challenging for younger people, who may be employed and/or raising children, as it marks the beginning of a life‐changing disease with significant psychosocial implications.^7^ Best practice for assessment, diagnosis, treatment, and care vary across services and locations, including referral patterns and application of diagnostic criteria, influenced by limitations in available diagnostic tests and resources.^8^ Research has identified that models of quality dementia care commonly include early assessment and diagnosis; access to appropriate interventions; a multidisciplinary team approach; person‐centered care; psychosocial and emotional support; and caregiver education and support.^9^

In Australia, dementia diagnoses are typically made by specialists (e.g., neurologist, psychiatrist, and geriatrician) or multidisciplinary cognition and memory clinics, accessed via referral from primary care. Some specialty YOD diagnostic clinics exist, often concentrated in metropolitan areas and linked to research centers, though they may accept referrals from across the state or even interstate. Australia currently lacks clear guidelines for diagnosing and caring for individuals with YOD. However, Australian Memory and Cognition Clinic Guidelines consider people suspected of YOD a high priority and recommend that they be offered an assessment within 30 days of referral.^10^ Australia has a universal healthcare system (Medicare), which provides rebates for a range of health services; however, some tests, treatments, and specialist consultations may incur out‐of‐pocket costs, either partially or in full.

An international Delphi study by O'Malley and colleagues identified and generated consensus statements regarding best practice diagnostic assessments for people with YOD.^11^ A follow‐up study by Lau and colleagues, which aimed to investigate the consensus of these statements among clinicians based in Australia, found similar agreement to the international study.^12^ This confirmed broad international agreement on what should constitute diagnostic assessments for YOD; in particular, the importance of a systematic approach to clinical history‐taking and physical examination to assess key features across a wide range of possible presentations. However, it did not consider nuances specific to Australia that can impact how diagnoses are made, such as its vast geography, with a significant proportion of people living in rural and remote areas, and its multicultural population, including Aboriginal and Torres Strait Islander peoples and culturally and linguistically diverse (CALD) communities. For instance, rural and regional residents may face long wait times for specialist appointments or must travel substantial distances to metropolitan areas to receive care due to lack of available services locally.^13^ While previous research has separately examined lived experiences or clinician perspectives, no study has yet triangulated the perspectives of all stakeholders involved in the diagnostic process to identify what works well and where improvements are needed and are feasible.

### 1.1 Aims

This study aimed to describe the experience of the diagnostic process for YOD in Australia, from symptom onset and initial consultation to diagnosis, exploring both challenges and positive aspects from the perspectives of people with lived experience and clinicians.

**RESEARCH IN CONTEXT**

1. **Systematic review**: The authors reviewed the literature primarily using PubMed. The path to diagnosing younger‐onset dementia is often marked by delays and misdiagnoses. Australia currently lacks clear guidelines for diagnosing this population. International experts agree on the importance of a systematic approach to clinical history‐taking and physical examination to identify key features across diverse presentations.
2. **Interpretation**: Our findings highlight both successes and areas for improvement, drawing from key stakeholders. While people in non‐metropolitan areas report better access to specialist clinics, long travel distances can be a barrier. Diagnosis is challenging due to clinical uncertainty, which can lead to misdiagnoses of psychiatric conditions. Diagnosis‐focused services help streamline the process. Experiences with diagnosis delivery vary, influenced by individual preferences and readiness.
3. **Future directions**: The manuscript proposes recommendations to optimize the diagnostic process, including implementing a hub‐and‐spoke model to expand specialist access, providing clinician training, establishing clear referral guidelines, and raising public awareness.

## 2 METHODS

### 2.1 Design

This is a sub‐study of the Joint Solutions Project, which was commissioned by the Australian Government Department of Social Services to develop a best‐practice system of care for individuals with YOD and their families. The study used a convergent mixed‐methods cross‐sectional approach, combining quantitative and qualitative research methods. Data were collected through surveys with multiple‐choice and open‐text responses, as well as focus group discussions. The comprehensive methodology of the Joint Solutions Project and overview of the service landscape at the time of the study are described in detail elsewhere^14^, ^15^ and outlined briefly here. Ethics approval was obtained from the University of Melbourne Human Research Ethics Committee (ID #28541).

### 2.2 Participants

This study included three of the four key stakeholder groups targeted for data collection in the broader project: (i) persons with YOD, (ii) their informal caregivers (e.g., family members), and (iii) clinicians involved in the diagnosis or care of persons with YOD in Australia, including general practitioners (GPs), medical specialists, and allied health practitioners. The fourth stakeholder group, community service providers, was not included in this study as they are primarily involved post‐diagnosis. In this study, stakeholder groups (i) and (ii) are collectively referred to as individuals with lived experience. Although GPs fall under stakeholder group (iii), they completed a separate survey and are reported as a distinct group in the quantitative analyses. However, they participated in clinician focus groups and are therefore included under the broader clinician category in the qualitative analyses. Participants self‐identified for inclusion and were recruited through nationwide advertising to professional and community groups. Those who completed the surveys were invited to express their interest in participating in focus groups to further explore themes around gaps, barriers, and positive aspects in the pathways to care for YOD. Of the 143 participants who expressed interest, 75 were invited to participate in focus groups, with efforts to balance gender, location (by state/territory and rurality), and role or relationship to a person with YOD. Of the 75 invited, 47 (62.7%) attended the focus groups. Non‐participation primarily resulted from scheduling challenges or last‐minute withdrawals due to personal circumstances (e.g., illness).

### 2.3 Materials

Surveys were developed based on existing literature and in consultation with a working party from the Australian YOD Special Interest Group network. The questions covered the entire care pathway from pre‐diagnosis to palliative care. This sub‐study focuses specifically on the pre‐diagnosis and diagnosis sections of the questionnaires. This included diagnostic pathways (e.g., referral processes and tests) and knowledge of dementia types (for GPs), as well as waiting times and specialists involved (for people with lived experience). Clinicians and people with lived experience were asked about the diagnostic assessments performed and their experience with diagnosis delivery (e.g., information provided and further referrals). Some sections contained statements that respondents rated on a five‐ or seven‐point Likert scale (i.e., strongly disagree to strongly agree, and not at all important to absolutely essential, respectively). For instance, in the clinician questionnaire, respondents were asked to rate statements related to diagnostic assessments, adapted from previous works,^11^, ^12^ based on their perceived importance in the context of a YOD diagnosis. The surveys for specialist clinicians and caregivers took approximately 30 minutes to complete, while those for GPs and people with YOD took approximately 5–10 minutes, as they were kept brief—acknowledging GPs’ time constraints and accommodating potential cognitive difficulties in people with YOD—by focusing only on core aspects of the diagnostic process.

Focus groups followed a semi‐structured format, with the interview schedule developed based on survey responses to facilitate a more contextual and detailed discussion.^14^ Some prompts were sent to participants in advance to allow reflection ahead of time (see Appendix 1), and PowerPoint slides were used to guide the discussion. Questions were tailored to each stakeholder group, reflecting their distinct roles and the scope of their respective survey content.

### 2.4 Data collection

Surveys were open for responses from 27 March to June 30, 2024 via the REDCap survey platform. Informed consent was obtained from all participants and responses were anonymous. Participants were eligible to enter a draw to win one of five $100 gift vouchers.

Ten focus groups were conducted; three for caregivers, three for clinicians, two for people with YOD, and two for community service providers. Each focus group comprised up to eight participants (fewer for those with lived experience to allow more time for each participant to respond to questions). Each focus group was facilitated by two members of the Joint Solutions leadership committee, with at least one person relevant to the respective stakeholder group (e.g., a clinician facilitating the clinician group). The sessions were conducted via the Zoom platform between 6 June and July 8, 2024 and lasted 60 minutes each. Focus group participants received a $100 gift voucher as compensation for their time.

### 2.5 Data analysis

Survey results were analyzed quantitatively using Jamovi software.^16^ Missing data from incomplete responses were excluded from the analysis, including the removal of participants who only completed the demographics section of the survey. Some parts of the surveys branched depending on the respondent's answers, meaning that participants only answered questions relevant to their roles and experiences. Descriptive statistics were computed, and, where applicable, comparison statistics were conducted using parametric analyses (i.e., χ2 tests to compare categorical data, t‐tests/analysis of variance [ANOVA] to compare continuous data). For clarity and ease of analysis, some variables were collapsed, such as by grouping Likert scale responses into three categories (e.g., Agree vs. Disagree vs. Neutral). The percentages presented in the results are based on the total number of respondents for each specific question.

Audio recordings of the focus groups were transcribed verbatim and analyzed using NVivo software version 14.^17^ All transcripts were de‐identified prior to analysis. Transcripts were analyzed together with open‐text responses from the surveys, using an inductive (i.e., data‐driven) thematic analysis with a critical realist grounded theory approach. This approach combines elements of critical realism, which posits that experience is constructed through, and influenced by, language and socio‐political‐cultural context, with grounded theory, a method for building theories from data. The goal is to understand not only what is happening, but also why and how, by considering the structural conditions that shape people's experiences.^18^ Thematic analysis involved six recursive phases: data familiarization, generating initial codes, searching for themes, reviewing and refining themes, defining and naming themes, and producing a report.^19^ Three authors (P.T., C.B., and D.S.) met regularly to review codes and preliminary themes and revise them to generate the final themes, which were then discussed with the leadership committee for sense‐checking. The full qualitative data analysis is reported elsewhere.^14^ This paper will report only the aspects relevant to the diagnostic process.

After completing the statistical and thematic analyses, we used methodological triangulation to integrate findings and develop a more comprehensive and nuanced understanding of the diagnostic experience. This involved comparing the extent to which the two datasets converged, complemented, or diverged. First, we cross‐referenced findings across datasets to identify alignment in key topics and experiences. Second, we assessed whether findings showed convergence (agreement), complementarity (related but distinct insights), or divergence (conflicting perspectives). This integration produced a synthesized dataset that enabled deeper interpretation and reflection. The triangulated findings formed the basis of the results presented in this paper. Relevant extract samples were selected to represent qualitative findings from both focus group discussions and open‐text survey responses.

## 3 RESULTS

A total of 313 participants from across Australia completed the questionnaires (Table 1 outlines their demographic information, for a complete summary refer to Loi et al.).^15^ People with lived experience represented 45% of the participants. Seventy‐one percent of caregivers were spouses or partners, while 18% were adult children. Most respondents were from Victoria and lived or worked exclusively in metropolitan areas. The majority of GPs (71%) had been practicing for more than 10 years. Other clinicians had an average of 13.1 years of experience working with people with YOD (SD = 9.6). On average, allied health clinicians saw 6 patients with YOD per month (SD = 8.8), medical clinicians saw 5 (SD = 4.8), and nurses saw 2 (SD = 1.7). Lived experience respondents predominantly reported a diagnosis of Alzheimer's disease or frontotemporal dementia (see Table 2). The average age at symptom onset was 55.8 years (SD = 4.8, range 39–65), and the average age at diagnosis was 57.9 years (SD = 5.4, range 40–70). Demographic information for the sub‐sample that participated in the focus groups is provided in Appendix 2.

**TABLE 1:** Demographic information for the four groups of respondents.

Parameter	YOD *n *= 33	Caregiver *n *= 105	GP *n *= 7	Specialist clinician *n *= 86
Female	17 (52%)	78 (74%)	6 (86%)	64 (74%)
Majority age range (years)	56–70 (79%)	56–65 (49%)	41–50 (43%)	41–50 (42%)
Non‐Caucasian	1 (3%)	2 (2%)	0	13 (15%)
State/territory				
ACT	4 (12%)	13 (13%)^a^	0	3 (3%)
NSW	10 (30%)	19 (18%)^a^	0	12 (14%)
NT	1 (3%)	2 (2%)^a^	0	3 (3%)
QLD	4 (12%)	7 (7%)^a^	0	3 (3%)
SA	2 (6%)	4 (4%)^a^	2 (29%)	5 (6%)
TAS	1 (3%)	1 (1%)^a^	1 (14%)	3 (3%)
VIC	10 (30%)	45 (43%)^a^	4 (57%)	42 (49%)
WA	1 (3%)	14 (13%)^a^	0	15 (17%)
Metropolitan^b^	15 (56%)		5 (71%)	63 (73%)
Discipline				
Neurology	–	–	–	9 (11%)
Psychiatry	–	–	–	12 (14%)
Geriatrics	–	–	–	14 (16%)
Other medical	–	–	–	1 (1%)
Neuropsychology	–	–	–	16 (19%)
Speech pathology	–	–	–	7 (8%)
Occupational therapy	–	–	–	6 (7%)
Other allied health	–	–	–	11 (13%)
Nursing	–	–	–	10 (12%)

Abbreviations: ACT, Australian Capital Territory; NSW, New South Wales; NT, Northern Territory; QLD, Queensland; SA, South Australia; TAS, Tasmania; VIC, Victoria; WA, Western Australia; YOD, person with young‐onset dementia.

^a^ Where the person with YOD lives (who the caregiver is providing support for).

^b^ Residing or working solely in metropolitan areas.

**TABLE 2:** Dementia subtypes among respondents with lived experience.

	YOD	Caregiver
Parameter	*n *= 33	*n *= 105
Alzheimer's dementia	11 (33%)	42 (40%)
Posterior cortical atrophy		8 (8%)
Frontotemporal dementia	8 (24%)	37 (35%)
Behavioral variant	5	21
Language variant	3	11
Lewy body disease	6 (18%)	5 (5%)
Vascular dementia	2 (6%)	1 (1%)
Mixed dementia	4 (12%)	7 (7%)
Other	1 (3%)	3 (3%)
Do not know^a^	1 (3%)	2 (2%)

Abbreviations: Caregiver, dementia information provided for those they were caring for; YOD, person with young‐onset dementia.

^a^ Response selected by participants, which may indicate that no specific subtype was communicated at diagnosis; however, the exact reason is unknown.

While 70% of caregivers reported that a diagnosis of dementia was provided within 12 months of presenting to the GP, 5% reported that it took more than 4 years. Over half (54%) of people with YOD felt they were kept updated throughout the diagnostic assessments. Additionally, caregivers living in nonmetropolitan areas reported increased likelihood of accessing specialist YOD or memory clinics compared to those living in metropolitan areas (χ2(1) = 7.62, p = 0.006). However, qualitative responses suggested that this may come at the cost of long travel distances.

We have geographical barriers in that services that involve neuropsychology for assessment are based in Hobart only. We are open statewide, but people have to be able to travel. (Clinician)

### 3.1 GP referral process

All the GP respondents (*n* = 7) were at least fairly confident in knowing where to refer to pursue a YOD diagnosis, with 63% saying they would refer to a neurologist and 50% to a cognition and memory clinic. In contrast, only 51% of caregivers believed their GP was aware of YOD, and 61% thought the GP knew where to refer for further investigations. While 57% of GPs agreed that they were able to differentiate YOD from a psychiatric condition, 43% disagreed. Similarly, 43% agreed that they were able to differentiate YOD from a non‐psychiatric condition, while 43% disagreed. None of the GPs reported being *completely confident* in recognizing any dementia subtype. While 43%–57% felt *fairly confident* in recognizing Alzheimer's, vascular, and alcohol‐related dementia, there was a notable lack of confidence in recognizing posterior cortical atrophy, variants of frontotemporal dementia, and Lewy body disease. Forty‐three percent of GP respondents were at least fairly confident in using bedside cognitive screening tools, with the Mini‐Mental State Examination (MMSE) being the most popular choice endorsed by 75% of respondents. However, some GPs qualitatively noted the need for more sensitive screening tools to detect cognitive and behavioral changes, including guidance on the right questions to ask when exploring symptoms.

My experience in YOD is that the screening tools are not always sensitive enough. It is the rest of the history and assessment that leads to the differential diagnosis, and the cognitive screening (e.g., MMSE) can remain normal for some time. (GP)

Eighty‐six percent of GPs knew where to refer for brain imaging, with most referring for computerized tomography (CT; 71%) and magnetic resonance imaging (MRI; 57%), and only one person referring for positron emission tomography (PET). All respondents indicated that imaging would typically be completed within 3 months of referral. Endorsed barriers to referral included costs to patients, lack of Medicare rebates for GP referrals, and not knowing where to refer for imaging.

### 3.2 Assessments

Clinicians involved in the diagnostic process rated the perceived importance of various aspects of history‐taking and diagnostic assessments for YOD. Statements that achieved high agreement on importance, defined as those with at least 80% of respondents rating them as “very important” or above (i.e., including “extremely important” and “absolutely essential”),^11^ are presented in Table 3. Of the total 31 statements, 19 achieved high agreement (e.g., obtaining collateral history, asking about changes in personality, behavior, and language). Conversely, statements related to the following investigations had less than 50% agreement on their importance for YOD diagnosis: cerebrospinal fluid biomarkers, amyloid imaging, genetic testing, and inquiries about law‐breaking activities (see Appendix 3 for a complete list of clinician‐rated statements and agreement rates).

**TABLE 3:** Statements on history‐taking and diagnostic assessments with high agreement on importance.

Statement	Agreement rate
To ask an informant (e.g., spouse) for a collateral history	99%
To ask about changes in behavior and personality (e.g., loss of empathy, loss of motivation, disinhibited behavior, change in food preferences)	98%
To ask about changes in language and communication abilities	97%
To ask whether there has been any changes in activities of daily living (e.g., food preparation, managing finances)	95%
To understand the symptom type and mode of onset	94%
To enquire about changes in physical health	92%
To order structural neuroimaging (i.e., MRI, CT)	91%
To conduct a cognitive screening assessment	90%
To consider previous medical conditions which may be linked with dementia (e.g., multiple sclerosis, rheumatoid arthritis, HIV, SLE)	88%
To understand the patient's educational and occupational history	87%
To take a thorough psychiatric history (including past and present)	87%
To ask about developmental history (e.g., learning difficulties)	86%
To obtain a complete medical history (including cardiovascular risk factors, autoimmune conditions, infections, etc.)	86%
To ask about sleep	86%
To understand the patient's life history and ask about stressful life events	86%
To take an alcohol and substance use history	85%
To ask if a first degree relative has/had dementia and their age of onset	84%
To examine for extrapyramidal features/praxis/motor skills	82%
To assess for previous head injuries	81%

Abbreviations: CT, computed tomography; HIV, human immunodeficiency virus; MRI, magnetic resonance imaging; SLE, systemic lupus erythematosus.

Clinicians qualitatively emphasized the importance of understanding the progression of symptoms, including their time course and chronicity. Many clinician participants also highlighted the value of obtaining information from both the individual and a reliable informant separately, as discrepancies between the two accounts can provide valuable diagnostic clues. Participants emphasized that it is essential to be mindful of the language used during these assessments, as patients may not fully understand neuropsychiatric jargon (e.g., “problems with organizing tasks, making decisions, or controlling impulses” rather than “executive dysfunction”). Information should be gathered from multiple sources, including previous investigations, and integrated into the current assessment to avoid redundancy. Participants felt that a thorough review of medical history, particularly neurological conditions, medication use, and infections, is crucial to identify other potential causes of cognitive impairment, some of which may be reversible. Clinician participants reflected that patients often prefer fewer investigations, thus efforts should be made to limit assessments to those considered necessary.

People with YOD have expressed the desire to not be assessed to death, so the case history can be vital in gathering information that may not be available via standardised assessments. (Clinician)

A majority of clinicians knew where to refer for various diagnostic procedures, with most tests completed three to 6 months after referral. For CT scans, 73% knew where to refer, and 64% reported that there would be no cost to the patient. For MRI scans, 70% knew where to refer, and 47% reported no cost to the patient. For PET scans, 66% knew where to refer, and 45% reported no cost to the patient. Only 47% knew where to refer for amyloid imaging, and 54% reported it would incur a cost to the patient. Common barriers endorsed for MRI, PET, and amyloid imaging included cost and practical challenges (e.g., travel distance). For single photon emission computed tomography scans, 51% knew where to refer, and 43% reported that there would be no cost to the patient, with inconclusive results being the main barrier to referral (28%). For lumbar punctures, 58% knew where to refer, and 44% reported no cost to the patient. Key barriers included patient concerns about invasiveness (40%) and practical challenges (30%). Among clinicians who were not neuropsychologists, 75% knew where to refer for neuropsychological testing. While nearly half (49%) of clinicians reported no cost to the patient, common barriers to accessing neuropsychological assessment included cost (45%), practical challenges (34%), and long wait times (30%). Forty‐one percent of referred patients were seen within 6 months, and 62% within 12 months. Ninety percent of caregivers reported that the person with YOD had access to brain imaging, although 59% incurred the cost. Similarly, 85% of caregivers reported access to cognitive or memory testing, with 54% covering the cost themselves.

### 3.3 Misdiagnoses

Forty‐two percent of respondents with YOD reported receiving prior diagnosis, most commonly psychiatric in nature, such as a mood disorder. Clinicians noted qualitatively that clinical uncertainty can make diagnosis challenging, as they must balance early detection with accuracy. In some cases, follow‐up appointments may be necessary to review the diagnosis, though this process may be frustrating for the person being assessed, who would have to endure uncertainty in the meantime. Additionally, mild cognitive impairment (MCI) was typically not considered a “useful” diagnostic label by either clinicians or lived experience respondents.

GPs need to know more about young‐onset dementia, as do some neurologists. We were lucky with our neurologists, but sadly have heard too many stories of people being misdiagnosed as being depressed and wasting years before a correct diagnosis. (Caregiver)

[We received a] diagnosis of mild cognitive impairment initially, [which] left us in a very gray area for 12‐24 months. (Caregiver)

### 3.4 Diagnosis delivery

Half of people with YOD (55%) agreed that the diagnosis was clearly communicated. Similarly, 52% of caregivers reported that the information they received was easy to understand, 48% had time to ask questions, but only 33% found the information helpful. Additionally, 26% reported that the health professional delivering the diagnosis was not sensitive to their needs. Almost 90% of caregivers received information about the relevant dementia subtype, but only 63% were provided with information about how their condition might change over time, including cognitive and behavioral changes. Only 46% of caregivers received information about prognosis.

The qualitative analyses revealed that the perceived quality of diagnosis delivery hinges on several factors: provision of adequate, personalized information and education, clarity of information provided, and demonstration of sensitivity and support.

Some empathy would have been great, rather than “Go home and get your life and affairs in order.” (Caregiver)

Some participants shared positive experiences, citing skilled clinicians, a thorough yet straightforward process, and timely diagnosis. Diagnosis‐focused services, such as memory clinics, appeared to help streamline the process.

I cannot praise enough each person we met and who worked with [person with YOD] at the next memory clinic. Each person was sensitive, kind, helpful, and friendly. Despite the awfulness of what was being contemplated, they made the process as positive and supportive as possible. (Caregiver)

However, a minority reported facing a lengthy journey to diagnosis and difficulties gaining access to diagnostic services.

I initially went to doctors in [the area] over 2 years ago, as I was concerned about my memory. I did not live in the area and so [the Health Service] wrote back to the doctor stating that I need to see specialists near me. I followed up with the doctors three times to see if they had referred me to relevant specialists but never got a response. I then went to a local doctor who made a referral to the relevant team (a cognition and memory clinic). I did not hear anything back from them for about 6 months, and so I followed up with them. They then booked me in very quickly and did all the cognitive tests and scans very fast. (Person with YOD)

Opinions were mixed regarding discussions about prognosis and disease progression; some participants wanted to know what to expect, while others found the topic too confronting.

### 3.5 Further referrals relating to diagnosis

#### 3.5.1 Genetic testing

None of the GPs felt confident discussing genetic testing with first‐degree relatives. Less than half of clinicians (40%) considered genotyping/genetic testing to be at least very important. Clinicians reported that they determined whether to refer for genetic testing as part of the diagnostic workup on a case‐by‐case basis (e.g., for younger patients or those with a family history of dementia), while also taking patient preferences into account. While genetic testing can be diagnostically informative, it can also feel confronting and overwhelming due to its implications for the next generation.

No, [the diagnosis] wasn't straightforward. And just to add, I think, mainly because a lot of the symptoms were masked and so for us it actually wasn't until genetic testing that actually confirmed it. (Caregiver)

Very cautious about unforeseen outcomes and the mental health implications [of genetic testing]. (Clinician)

Some participants suggested that genetic testing could be raised during the diagnostic assessment and revisited in follow‐up appointments. Involving a genetic counsellor in the process was also seen as valuable. However, many clinicians lack understanding of the genetic underpinnings of YOD, including the specific genes involved. This uncertainty, coupled with a lack of clear referral guidelines, makes it difficult to determine when to refer for genetic testing. Only 46% of clinicians reported knowing where to refer for genetic testing, and 41% were uncomfortable providing preliminary genetic counseling before referral. A quarter of caregivers (26%) reported access to genetic testing for the person with dementia. Of these, 81% received the testing at no cost, and half also had access to genetic counselling.

#### 3.5.2 Diagnosis‐related education

All clinicians agreed on the importance of providing information and education about caring for someone with dementia, and 85% reported they would provide this information themselves. However, 60% of caregivers disagreed or strongly disagreed that they had received such education. Additionally, 57% of GPs were unsure where to refer people with YOD and their caregivers for educational resources. Those who did receive education typically did so within 6 months of diagnosis, and found it helpful and YOD‐specific.

Diagnosis‐related education that participants called for included the science underpinning dementia, expected behavioral changes and management strategies, communication techniques, how to create an enabling environment, and available supports and services—including through Australian government social care funding programs such as the National Disability Insurance Scheme (NDIS) or Disability Support Pension (DSP). However, some recipients felt more tailored information was needed, such as details specific to the person's type of YOD, behaviors of concern at different stages of the illness, and support for younger caregivers based on their stage in life.

We found getting information out of GPs after the diagnosis was almost impossible. They basically palm you off to a specialist and say, “Ask them.” You can get into a specialist once every 6 months… No advice to get in touch with Dementia Australia, and no advice on how to go about an NDIS or DSP application… Better education at that level would be wonderful. (Caregiver)

#### 3.5.3 Research trials

Following a diagnosis, people with YOD and their caregivers are often referred to research trials. The majority of clinician respondents (81%) agreed or strongly agreed on the importance of providing information about these trials, while 45% of caregivers reported receiving such information. Some caregivers find participating in trials helpful for gaining knowledge and contributing to society.

The clinical researcher leading the research was the best source of information for our family, they were better than any medical professional and answered all our questions and pointed us in the right directions despite living in another state and not being a health professional rather a scientific researcher. (Caregiver)

However, a barrier to referrals is that some clinicians are unaware of available trials. If trials are introduced, participants emphasized the importance of considering timing and sensitivity, noting that raising research participation immediately after diagnosis may not be appropriate while people are still processing the news, and may pose a barrier to patient engagement.

There needs to be a level of sensitivity by the medical practitioner when discussing research involvement. Raising the topic of research shortly after giving the person their diagnosis of dementia is often perceived as very insensitive and prompted by self‐interest. Extra care and measured timing of such information needs to be carefully considered. (Clinician)

It was too soon after his diagnosis. He needed to process this as it was life changing. (Caregiver)

Participants also emphasized the importance of managing expectations and clarifying that research does not guarantee favorable outcomes. Some respondents appreciated having the autonomy to choose whether, when, and what research to participate in, while others faced barriers such as eligibility restrictions (e.g., medical comorbidities, age cut‐offs, severity of condition), reluctance to participate (e.g., anxiety/stress‐inducing, perceived invasiveness), or inability to engage (e.g., caregiver lacking time to take them to appointments).

## 4 DISCUSSION

This study examined the experience of obtaining a YOD diagnosis in Australia, aiming to highlight challenges and positive aspects. Our findings suggest a promising trend in the timeliness of YOD diagnoses, with most participants reporting a time to diagnosis of 6–12 months from presenting with symptoms to a GP. This represents a significant improvement compared to past Australian literature, which estimated a diagnostic delay of 3.5 to 5 years.^6^, ^20^ This improvement may be attributed to the increase in memory clinics and specialist diagnostic services, which are key in facilitating more streamlined and timely dementia diagnoses.^21^ However, barriers to accessing these services persist, especially pertaining to geographical disparities and underserved areas. Specialist availability is often concentrated in metropolitan areas and varies by state and territory.^13^, ^14^, ^22^ Interestingly, our study found that individuals living outside metropolitan areas were more likely to access specialist services. This could reflect referrals to statewide services in the absence of local specialists, albeit at the expense of having to travel long distances. To improve access to timely and accurate diagnoses, regardless of location, a potential solution is employing a hub‐and‐spoke model.^23^, ^24^ In such models, larger specialized centers collaborate with regional practices to facilitate sharing of expertise and resources. This approach can upskill clinicians while improving accessibility of care and patient outcomes.

Our study also raises concerns about the overall lack of awareness of YOD, both within the general community and among healthcare professionals, especially outside the small number of dementia care specialists. GPs in our study, a small group likely with pre‐existing interest in dementia, reported lower confidence in recognizing rarer and more complex forms of YOD, such as posterior cortical atrophy, frontotemporal dementia, Lewy body disease, and dementia secondary to Parkinson's disease. These conditions can be particularly challenging to identify due to their atypical presentations, which often overlap with or are mistaken for other conditions, including mental health disorders.^1^ In contrast, GPs reported higher confidence in recognizing more common dementias, such as Alzheimer's disease and vascular dementia, which have more well‐established presentations. Underdiagnosis is common in general practice, as memory loss typically triggers dementia investigations, but GPs may overlook impairments in other cognitive or behavioral domains.^21^ As GPs are often the first point of contact for people with memory concerns, it is crucial to provide them and other frontline clinicians with more training to recognize indicators of YOD. Clear guidelines and resources need to be developed to streamline the referral process to memory clinics and specialist services. For example, the United Kingdom (UK)‐based Young Dementia Network provides a guide to help GPs recognize symptoms of dementia in younger people. ^25^ Additionally, raising public awareness can help individuals better recognize symptoms and seek help earlier, as well as alleviate stigma and social isolation.^26^, ^27^

In terms of diagnostic assessments, 19 aspects were agreed upon as important, with clinicians emphasizing history‐taking and the information gathered during this process. Nearly unanimous agreement was reached on the importance of obtaining a collateral report from a reliable informant. Other key components included understanding the symptom type and mode of onset, particularly changes in behavior and personality, language and communication, as well as activities of daily living. These findings align with those of O'Malley et al.^11^ and Lau et al.,^12^ who also highlight the value of a structured and detailed history in diagnosing YOD, as understanding symptom progression is critical for accurate diagnostic formulation. Structural neuroimaging, cognitive screening, and physical examination were also deemed essential, underscoring the value of a comprehensive, multifaceted diagnostic approach. This study extends the findings of Lau et al.^12^ by including greater representation from across Australia, with clinicians from both rural and metropolitan areas. Additionally, this study highlights barriers to certain diagnostic assessments, such as the perceived invasiveness of certain investigations and their associated costs.

Some investigations were considered less central to diagnosing YOD, including cerebrospinal fluid biomarkers, amyloid imaging, and genetic testing. Considering YOD presents atypically, often with psychiatric symptoms, using investigations such as biomarkers to confirm or refute the diagnosis is recommended.^5^ These tools may be considered relatively new, with research still emerging, and some, like amyloid imaging, are currently only available in research settings and not in routine clinical practice.^5^, ^28^ Cerebrospinal fluid biomarkers, which have shown high sensitivity and specificity for Alzheimer's disease,^29^ are now included in the new consensus criteria for preclinical Alzheimer's diagnosis.^30^ While valuable, these investigations may require further development, increased awareness, and broader accessibility to be more widely implemented in the diagnostic process for YOD in Australia.

Upon receiving a YOD diagnosis, caregivers expressed a need for guidance on what to expect during the different stages of the condition and where to seek support, both for practical caregiving and emotional wellbeing. The literature supports the importance of educating caregivers and support networks about YOD to ensure that they are well‐prepared for the challenges ahead.^31^ Tailored and targeted resources for caregivers may help alleviate stress and optimize quality of life, as high caregiver burden is linked to poor outcomes for both caregiver and the person with YOD, including premature placement in residential care that may not be age‐appropriate for individuals with YOD.^32^

Another key aspect of the diagnostic process highlighted in our findings is the importance of considering both the information delivered and the manner in which it is communicated to patients and their families. Clinician sensitivity and supportiveness are highly valued, especially when delivering a diagnosis that confirms a life‐changing condition. Our findings align with previous research, which emphasizes the critical role of interpersonal factors in healthcare interactions, noting that even brief negative encounters can have lasting effects on future healthcare trajectories.^33^ People with lived experience have also expressed a desire for a follow‐up appointment offering emotional support soon after diagnosis,^27^ and this might provide an opportunity for clinicians to “check in,” providing psychoeducation if requested and to answer questions arising with time. Everyone responds differently to a YOD diagnosis–some with devastation or shock, others with denial or avoidance, and some with relief.^13^ These responses can also change over time, presenting a challenge for clinicians in determining the right moment to provide information or refer to research trials. Nonetheless, it is an important factor to consider.

This study's mixed‐methods approach provides the advantage of combining structured survey responses, which facilitate quantifiable analysis, with in‐depth narratives of personal experiences, allowing for a more comprehensive understanding of the YOD diagnostic process. The use of a critical realist grounded theory approach enables the exploration of both individual experiences and the underlying social structures and mechanisms shaping the diagnostic process. Several limitations should be considered when interpreting the findings of this study. First, the very small number of GP respondents limits the generalizability of the results, as the sample may not fully represent the broader population of GPs working in Australia who encounter patients with possible YOD. Additionally, the short recruitment period may have impacted the number of participants and limited the diversity of responses. There was unequal representation from different states and territories, which limited our ability to compare and contrast differences across jurisdictions, relevant given regional differences in healthcare services. There was also a lack of diversity in the sample, particularly with respect to CALD groups and Aboriginal and Torres Strait Islander communities, which may affect the applicability of these findings to these populations. Study participants may be subject to self‐selection bias, with participating clinicians likely more engaged in YOD care, and lived experience participants potentially involved in advocacy or support networks. Further research with a larger, more diverse sample is needed to better capture the perspectives of all relevant groups in Australia.

Future research should focus on evaluating existing, high‐performing diagnostic services to identify features that can be translated to other regions or healthcare settings. There is a need to develop new services to meet the growing demand for YOD care, including exploring the feasibility of a hub‐and‐spoke model. Additionally, developing resources to guide referrals and assessments, along with workforce training programs, would be important. Further investigation into GP perspectives on recognizing YOD and making referrals could inform training needs and improve early diagnosis and care navigation. Finally, research is needed to explore the experiences of disadvantaged groups, such as those experiencing homelessness or incarceration, to facilitate better access to diagnosis and care in these populations.

## CONFLICT OF INTEREST STATEMENT

The authors declare no conflict of interest. Author disclosures are available in the Supporting Information.

## CONSENT STATEMENT

All human subjects provided informed consent to participate in this study.

## Supporting information



Supporting Information

Supporting Information
