# Supplementary material for: The road to young‐onset dementia diagnosis: Findings from the Joint Solutions Project
Source: Alzheimers Dement. 2025 Aug 12;21(8):e70538. doi: 10.1002/alz.70538 (PMC12340422; doi:10.1002/alz.70538)
Supplement: Supplementary file 2 — Supporting Information [file ALZ-21-e70538-s001.docx]

**Appendix 1. Diagnosis-related questions sent to participants in preparation for focus group sessions.**

1. **People with YOD**
2. What was it like when you first realised you were having symptoms?
3. What was it like to seek professional help and diagnosis?
4. How would you describe the process of the diagnosis?
5. What worked well or did not work well?
6. **Caregivers**
7. Can you briefly share what actions you took when you first realised your family member had symptoms of dementia?
8. Yes or no - were the steps to formalise a diagnosis made clear to you? If yes by who?
9. During this time what support or advice was available to you?
10. **Clinicians**
11. What makes identifying young-onset dementia and initiating the diagnostic process challenging?
12. Are you familiar with services that are able to assist with the diagnostic process? For those of you who work in such a service, is the referral process well known to others?

**Appendix 2. Demographic characteristics of focus group participants.**

|  | **YOD**  *n*=10 | **Caregiver** *n*=13 | **Clinician**  *n*=14 |
| --- | --- | --- | --- |
| **Female** | 6 (60%) | 7 (54%) | 11 (79%) |
| **State/Territory**  ACT  NSW  NT  QLD  SA  TAS  VIC  WA | 2 (20%)  2 (20%)  1 (10%)  2 (20%)  1 (10%)  1 (10%)  1 (10%)  0 (0%) | 1 (8%)  3 (31%)  0 (0%)  2 (15%)  2 (15%)  0 (0%)  3 (31%)  2 (15%) | 1 (7%)  3 (21%)  2 (14%)  1 (7%)  2 (14%)  1 (7%)  3 (21%)  1 (7%) |
| **Metropolitan^** | 6 (60%) | 8 (62%) | 10 (71%) |

YOD: person with young-onset dementia; ACT: Australian Capital Territory; NSW: New South Wales; NT: Northern Territory; QLD: Queensland; SA: South Australia; TAS: Tasmania; VIC: Victoria; WA: Western Australia
^residing or working solely in metropolitan areas

**Appendix 3. Clinician agreement rates on statements related to history-taking and diagnostic assessment practices.**

| **Statement** | **Agreement Rate** |
| --- | --- |
| To ask an informant (e.g., spouse) for a collateral history | 99% |
| To ask about changes in behaviour and personality (e.g., loss of empathy, loss of motivation, disinhibited behaviour, change in food preferences) | 98% |
| To ask about changes in language and communication abilities | 97% |
| To ask whether there has been any changes in activities of daily living (e.g., food preparation, managing finances) | 95% |
| To understand the symptom type and mode of onset | 94% |
| To enquire about changes in physical health | 92% |
| To order structural neuroimaging (i.e., MRI, CT) | 91% |
| To conduct a cognitive screening assessment | 90% |
| To consider previous medical conditions which may be linked with dementia (e.g., multiple sclerosis, rheumatoid arthritis, HIV, SLE) | 88% |
| To understand the patient’s educational and occupational history | 87% |
| To take a thorough psychiatric history (including past and present) | 87% |
| To ask about developmental history (e.g., learning difficulties) | 86% |
| To obtain a complete medical history (including cardiovascular risk factors, autoimmune conditions, infections, etc.) | 86% |
| To ask about sleep | 86% |
| To understand the patient’s life history and ask about stressful life events | 86% |
| To take an alcohol and substance use history | 85% |
| To ask if a first degree relative has/had dementia and their age of onset | 84% |
| To examine for extrapyramidal features / praxis / motor skills | 82% |
| To assess for previous head injuries | 81% |
| Assessment of gait and balance | 79% |
| Assessment of eye movements | 75% |
| Neuropsychological assessment | 75% |
| Dementia routine blood screen | 74% |
| Conducting a physical examination | 74% |
| To conduct a mental state examination | 71% |
| Assessment of cerebellar signs | 70% |
| Functional neuroimaging (i.e., SPECT, PET) | 69% |
| To ask about any recent offences or law-breaking activities (e.g., shoplifting) | 48% |
| Genotyping or genetic testing | 41% |
| Amyloid imaging | 39% |
| Cerebrospinal fluid biomarkers | 38% |
